# Supervised Machine Learning Empowered Multifactorial Genetic Inheritance Disorder Prediction

**DOI:** 10.1155/2022/1051388

**Published:** 2022-05-31

**Authors:** Taher M. Ghazal, Hussam Al Hamadi, Muhammad Umar Nasir, Atta-ur-Rahman, Mohammed Gollapalli, Muhammad Zubair, Muhammad Adnan Khan, Chan Yeob Yeun

**Affiliations:** ^1^School of Information Technology, Skyline University College, Sharjah 1797, UAE; ^2^Network and Communication Technology Lab, Center for Cyber Security, Faculty of Information Science and Technology, Universiti Kebangsaan, Malaysia 43600, Malaysia; ^3^Center for Cyber Physical Systems, Khalifa University, Abu Dhabi, 127788, UAE; ^4^Riphah School of Computing & Innovation, Faculty of Computing, Riphah International University Lahore Campus, Lahore 54000, Pakistan; ^5^Department of Computer Science, College of Computer Science and Information Technology, Imam Abdulrahman Bin Faisal University, P.O. Box 1982, Dammam 31441, Saudi Arabia; ^6^Department of Computer Information Systems, College of Computer Science and Information Technology, Imam Abdulrahman Bin Faisal University, P.O. Box 1982, Dammam 31441, Saudi Arabia; ^7^Faculty of Computing, Riphah International University, Islamabad 45000, Pakistan; ^8^Pattern Recognition and Machine Learning Lab, Department of Software, Gachon University, Seongnam, Gyeonggido 13120, Republic of Korea

## Abstract

Fatal diseases like cancer, dementia, and diabetes are very dangerous. This leads to fear of death if these are not diagnosed at early stages. Computer science uses biomedical studies to diagnose cancer, dementia, and diabetes. With the advancement of machine learning, there are various techniques which are accessible to predict and prognosis these diseases based on different datasets. These datasets varied (image datasets and CSV datasets) around the world. So, there is a need for some machine learning classifiers to predict cancer, dementia, and diabetes in a human. In this paper, we used a multifactorial genetic inheritance disorder dataset to predict cancer, dementia, and diabetes. Several studies used different machine learning classifiers to predict cancer, dementia, and diabetes separately with the help of different types of datasets. So, in this paper, multiclass classification proposed methodology used support vector machine (SVM) and K-nearest neighbor (KNN) machine learning techniques to predict three diseases and compared these techniques based on accuracy. Simulation results have shown that the proposed model of SVM and KNN for prediction of dementia, cancer, and diabetes from multifactorial genetic inheritance disorder achieved 92.8% and 92.5%, 92.8% and 91.2% accuracy during training and testing, respectively. So, it is observed that proposed SVM-based dementia, cancer, and diabetes from multifactorial genetic inheritance disorder prediction (MGIDP) give attractive results as compared with the proposed model of KNN. The application of the proposed model helps to prognosis and prediction of cancer, dementia, and diabetes before time and plays a vital role to minimize the death ratio around the world.

## 1. Introduction

Dementia, a degenerative brain illness, is a significant health issue in terms of global health, public health, and population health [1]. Understanding the development of dementia illness and aiding early identification of dementia have been recent focal points of study in the disciplines of neuroimaging and genetics [2]. Numerous genome-wide association studies have also been undertaken since 2007 to discover genetic variations such as single nucleotide polymorphisms that are linked to dementia [3]. Researchers have continued to make significant discoveries in the interdisciplinary disciplines of machine learning, neuroimaging, genomics, and dementia diagnosis and prediction as artificial intelligence tools have improved [4, 5]. Recent advancements in artificial intelligence (AI) technology, notably machine learning approaches, have demonstrated their usefulness in health-related and genetic medicine applications [6, 7]. Under specific environmental settings, biological characteristics are the consequence of interactions between gene sequences and gene interactions.

The machine learning model is appropriate for investigating the link between these variables and phenotype. Through machine learning of genomes, genome machine learning (GML) investigates the connection among genetic variants and characteristics. Although genome-wide association study (GWAS) is utilized to detect correlations between single nucleotide variants and cancer, it depends on linkage analysis to find sick genes and necessitates more intimate segregated locations [8, 9]. Diabetes mellitus is a chronic illness characterized by persistent hyperglycemia induced by a variety of factors. The primary cause is a deficiency in insulin secretion. Typical symptoms include polyuria, polydipsia, polyphagia, and weight loss, which may be accompanied by skin itching. Long-term carbohydrate, lipid, and protein metabolism problems can also result in several chronic consequences, including chronic progressive illness, hypofunction, and tissue failure, as well as organs such as the eyes, kidneys, nerves, heart, and blood vessels.

Large quantities of data hide important information and insights in the age of big data. A significant quantity of data filtered by relevant data sources is merged into a data set for data mining to predict dementia, cancer, and diabetes. Following that, users may use machine learning algorithms to classify and analyze this dataset. This not only allows patients to avoid and cure dementia, cancer, and diabetes at an early stage through prediction but it also saves a significant amount of time and money. This paper employs a variety of algorithms to train an integrated data set before proposing an algorithm that may utilize the medical history of an early genetic problem to predict dementia, cancer, and diabetes. Major purpose of this study is to get efficient prediction results for cancer, dementia, and diabetes using different machine learning algorithms and test machine learning model performance using numerous statistical parameters. With the help of improvised results, medical field will get major benefits from this study and play their pivot role in serving for people.

## 2. Related Work

In recent years, two large multicenter studies have been conducted to discover biomarkers for early diagnosis of dementia and the development of Medical Council of India (MCI) to dementia: the auxiliary nursing midwifery, which is located in Europe, and the dementia disease neuroimaging initiative, which is based in the United States. Furthermore, at national center for biotechnology information gene expression omnibus, a substantial quantity of publicly available gene expression data on dementia has been given [10]. As a result, numerous research studies, particularly gene expression-based investigations, have been published to identify the informative genes linked to dementia. The brain net study examined 113 well-characterized postmortem brain tissue samples, resulting in the identification of 21 dysregulated genes in dementia [11]. A study of 87 brain tissue samples by Liang et al. found that the genes encoding the subunits of mitochondrial components were substantially lower expressed in the brain tissues of dementia patients. Xu et al. [12] discovered that an early change in protein1 might. Cause dementia by examining the ribonucleic acid expression of brain tissues from dementia patients. Although numerous studies utilizing gene expression data have found significant patterns, the majority of the gene expression data was collected from biopsies or autopsy-based and patients data samples, which makes extrapolation to clinical settings challenging. Only a few research [13] utilized blood expression data to identify important genes associated with dementia or to predict early dementia. Cooper et al. [13] presented research that included 186 dementia patients and 204 controls from three different data sets, indicating that progranulin expression levels in the blood are higher in dementia. Tae-WooKim et al. [14] created an operational lung cancer decision tree. The occupational safety and health researchers institute recorded 153 instances of lung cancer between 1992 and 2007. The goal was to evaluate if the condition was acknowledged as lung cancer connected to age, gender, years of smoking, histology, industry size, delay, work hours, and exposure of independent factors. The characterization and relapse test concept are utilized along the route of word-related cell degradation markers in the lungs. The greatest signal of the lungs cancer detection model was its introduction to well-known lung disease experts. In 2014, Maciej Ziba et al. [15] presented powered SVM, which is devoted to addressing imbalanced outcomes. For unequal data, the suggested approach coupled the benefits of employing set classifiers with cost-sensitive support vectors. A technique for extracting choices from the improved SVM was also provided. The effectiveness of the suggested method was then assessed by comparing the performance of the imbalanced data with that of other algorithms. Finally, in lung cancer patients, enhanced SVM was utilized to estimate life expectancy following surgery. Numerous techniques, including classic machine learning methods [16], such as support vector machine, decision tree (DT), logistic regression, and others, have recently been applied to predict diabetes. The authors in [17] proposed the linear discriminant analysis diabetes prediction method. The authors utilized linear discriminant analysis in this system to decrease dimensionality and extract features. To deal with the high-dimensional data sets, the authors in [18] built logistic regression-based prediction models for several type 2 diabetes prediction beginnings. The authors in [19] focused on hyperglycemia and employed support vector regression (SVR), a regression analysis issue, to predict diabetes. Furthermore, joint techniques are being used in an increasing number of research to increase accuracy [16]. The authors in [20] presented rotation forest, a novel ensemble technique that incorporates 30 machine learning algorithms. The authors in [21] suggested a machine learning technique that altered the SVM prediction criteria. In 2017, Kee Pang Soh et al. [22] created a cancer prediction model using SVM and applied their model on sequenced tumored DNA and achieved 77.7% prediction accuracy. In 2019, Javier De Velasco Oriol et al. [23] suggested a machine learning technique for dementia prediction using neuroimaging and their machine learning model achieved 75% classification accuracy. In 2013, BAssam Farran et al. [24] used four machine learning techniques such as linear regression, SVM, KNN, and multifactorial dimension reduction to predict diabetes, and their model achieved 81.3% highest prediction accuracy from SVM. In [25], researcher proposed feature extraction techniques with machine learning algorithms for the prediction of dementia. They measured the performance of their model with the help of different statistical parameters. In this study [25], researchers classify the dementia using different machine learning algorithms and achieved 83% testing accuracy. All previous researches used different limited data for single class classification with limited approaches of machine learning algorithms and feature extraction techniques and achieved lowest prediction accuracy.

Machine learning algorithms are frequently utilized to forecast diabetes, dementia, and cancer and achieve better outcomes. SVM and KNN are two prominent machine learning algorithms in the medical area, and they have high sorting power. SVM and KNN are two newly prominent machine learning methods that outperform others in many ways. In this paper, to predict diabetes, dementia, and cancer, the proposed model utilized a support vector machine and K-nearest neighbors using multifactorial genetic inheritance disorder data of sick patients and predicted multiclass diseases efficiently.

## 3. Research Methodology

**Figure 1:**
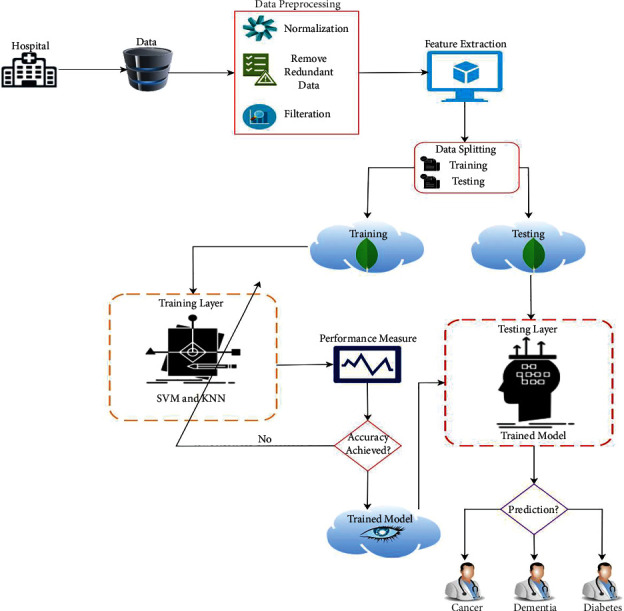
Proposed machine learning-based cancer, dementia, and diabetes prediction model from multifactorial genetic disorder.

The proposed machine learning-based cancer, dementia, and diabetes prediction model from multifactorial genetic disorder is shown in Figure 1. In the first phase, data are collected from hospital and stored in database; right after this step, the proposed model performed two steps; first is data preprocessing to normalize data using different normalization techniques and removing the duplicate data using different queries and in second step the proposed model performed correlation technique to extract high performed features for further training and testing step. So, the proposed model divided data into testing and training and stored in separate data clouds. In the second phase, the proposed model used machine learning algorithms to train data and check the model performance if the performance of trained achieve the benchmark than the trained model stored in cloud database otherwise retrain the models. In the final and third phase, the proposed model imported testing data from test data cloud and trained model from train cloud and performed testing queries to predict the cancer, dementia, and diabetes.

## 4. Dataset

The dataset in this research paper is obtained from open-source Kaggle [26]. This dataset consists of 2067 medical patients' history which is diagnosed with cancer, dementia, and diabetes. The total dataset consists of 32 independent attributes including, patient age, genes in mothers' side, inherited from father, maternal gene, no. of previous abortions, and so on and one dependent attribute.

Table 1 shows some of the dataset's attributes descriptions, 1 indicates “yes” and 2 indicates “no” and in gender attribute 3 indicates “ambiguous” gender.

**Figure 2:**
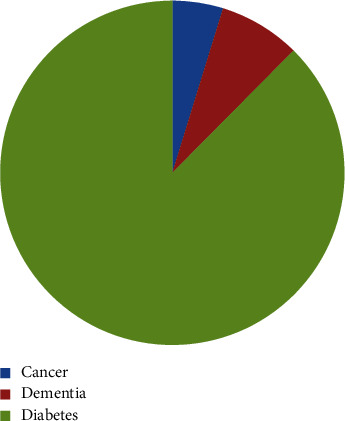
No. of patients from targeted classes.

Figure 2 shows the no. of patients from the targeted class and targeted class patients 1822, 152, and 93 from diabetes, dementia, and cancer, respectively.

## 5. Support Vector Machine

Support vector machine is a general linear predictor that uses supervised learning to perform three-class data classifications. Its decision limit for handling learning patterns is the maximum margin hyperplane [23]. Support vector machine calculates empirical risk using the pivot loss function and optimizes structural risk by including a regularization component in the solution system. It is a scarcity and robustness predictor [24].

The kernel technique, which is one of the most prevalent kernel learning methods, allows the support vector machine to execute a nonlinear sort.

SVM hypothesis is as follows:

(1) $$\begin{array}{c} F_{\theta }\left(q\right)=\left\{\begin{array}{cc} 1 & \text{if }\theta^{v}f>=0\\ 0 & \text{otherwise} \end{array}\right),\\ \theta^{v}f=\theta_{0}f_{0}+\theta_{1}f_{1}+\cdots +\theta_{z}f_{z}. \end{array}$$

SVM loss function is as follows:  

(2) $$\begin{array}{c} D\left(\theta \right)=k\left[\overset{z}{\underset{g=1}{\sum }}y^{\left(i\right)}{\text{Cost}}_{1}\left(\theta^{v}\left(f^{\left(g\right)}\right)+\left(1-y^{\left(g\right)}\right){\text{Cost}}_{\text{0}}\left(\theta^{v}\left(f^{\left(g\right)}\right)\right.\right)\right]. \end{array}$$

SVM regularized loss function is as follows:

(3) $$\begin{array}{c} D\left(\theta \right)=C\left[\sum_{g=1}^{z}\left[y^{\left(g\right)}{\text{Cost}}_{1}\left(\theta^{r}\left(f^{\left(g\right)}\right)+\left(1-y^{\left(g\right)}\right){\text{Cost}}_{0}\left(\theta^{T}\left(f^{\left(g\right)}\right)\right.\right.\right]\right.+\frac{1}{2}{\sum_{h=1}^{z}\theta_{h}^{2}}^{}. \end{array}$$

## 6. Simulation Results

In this study, MATLAB R2021 is used for simulation and predictions. The proposed model is used to train and test patients' data using machine learning techniques like SVM and KNN. In starting of the simulation proposed model, split the dataset (2067 instances) into 70% (1447 instances) for training and 30% (620 instances) for testing. After applying the proposed research model on the training dataset using both SVM and KNN machine learning algorithms, we get the SVM trained model and KNN trained model for prediction purposes. After the availability of the trained model, these trained models were used to predict cancer, dementia, and diabetes using the testing dataset. After that, we select the best prediction model by applying different statistical performance parameters like classification accuracy (CA), missclassification rate (MCR), sensitivity, specificity, F1-score, positive predicted value (PPV), negative predicted value (NPV), false positive ratio (FPR), false negative ratio (FNR), likelihood positive ratio (LPR), and likelihood negative ratio (LNR) on simulation results. Simulation results of the prediction proposed model are elaborated below with respect to the confusion matrix and different performance statistical parameters. In confusion matrix, ∂ represents true positive results, *µ* represents true negative results, Ø represents false positive results, and Ω represents false negative results.

(4) $$\begin{array}{c} \partial_{i}=\frac{\varphi_{i}}{\varrho_{i}}, \end{array}$$

where *φ* is for predicted class and *ϱ* for true class:

(5) $$\begin{array}{c} {µ}_{i}=\sum_{j=1}^{3}\left(\frac{\varphi_{i}}{\varrho_{j\neq i}}\right),\\ {Ø}_{i}=\sum_{j=1}^{3}\left(\frac{\varphi_{j\neq i}}{\varrho_{i}}\right),\\ \Omega_{i}=\sum_{j=1}^{3}\left(\frac{\varphi_{j\neq i}}{\varrho_{j\neq i}}\right),\\ \text{CA}=\frac{\varphi_{i}/\varrho_{i}+\sum_{j=1}^{3}\left(\varphi_{i}/\varrho_{j\neq i}\right)}{\varphi_{i}/\varrho_{i}+\sum_{j=1}^{3}\left(\varphi_{i}/\varrho_{j\neq i}\right)\;+\sum_{j=1}^{3}\left(\varphi_{j\neq i}/\varrho_{i}\right)+\sum_{j=1}^{3}\left(\varphi_{j\neq i}/v_{j\neq i}\right)},*100,\\ \text{CMR}=100-\left(\frac{\varphi_{i}/\varrho_{i}+\sum_{j=1}^{3}\left(\varphi_{i}/\varrho_{j\neq i}\right)}{\varphi_{i}/\varrho_{i}+\sum_{j=1}^{3}\left(\varphi_{i}/\varrho_{j\neq i}\right)\;+\sum_{j=1}^{3}\left(\varphi_{j\neq i}/\varrho_{i}\right)+\sum_{j=1}^{3}\left(\varphi_{j\neq i}/\varrho_{j\neq i}\right)}*100\right),\\ \text{Sensitivity}=\frac{\varphi_{i}/\varrho_{i}}{\varphi_{i}/\varrho_{i}+\sum_{j=1}^{3}\left(\varphi_{j\neq i}/\varrho_{j\neq i}\right)}*100,\\ \text{Specifity}=\frac{\sum_{j=1}^{3}\left(\varphi_{i}/\varrho_{j\neq i}\right)}{\sum_{j=1}^{3}\left(\varphi_{i}/\varrho_{j\neq i}\right)+\sum_{j=1}^{3}\left(\varphi_{j\neq i}/\varrho_{i}\right)}*100,\\ F1-\text{Score}=\frac{2\;\varphi_{i}/\varrho_{i}}{2\varphi_{i}/\varrho_{i}+\sum_{j=1}^{3}\left(\varphi_{j\neq i}/\varrho_{i}\right)+\sum_{j=1}^{3}\left(\varphi_{j\neq i}/\varrho_{j\neq i}\right)}*100,\\ \text{PPV}=\frac{\varphi_{i}/\varrho_{i}}{\varphi_{i}/\varrho_{i}+\sum_{j=1}^{3}\left(\varphi_{j\neq i}/\varrho_{i}\right)}*100,\\ \text{NPV}=\frac{\sum_{j=1}^{3}\left(\varphi_{i}/\varrho_{j\neq i}\right)}{\sum_{j=1}^{3}\left(\varphi_{i}/\varrho_{j\neq i}\right)+\sum_{j=1}^{3}\left(\varphi_{j\neq i}/\varrho_{j\neq i}\right)}*100,\\ \text{FPR}=100-\left(\frac{\sum_{j=1}^{3}\left(\varphi_{i}/\varrho_{j\neq i}\right)}{\sum_{j=1}^{3}\left(\varphi_{i}/\varrho_{j\neq i}\right)+\sum_{j=1}^{3}\left(\varphi_{j\neq i}/\varrho_{i}\right)}*100\right),\\ \text{FPR}=100-\left(\frac{\varphi_{i}/\varrho_{i}}{\varphi_{i}/\varrho_{i}+\sum_{j=1}^{3}\left(\varphi_{j\neq i}/\varrho_{j\neq i}\right)}*100\right),\\ \text{LPR}=\frac{\varphi_{i}/\varrho_{i}/\varphi_{i}/\varrho_{i}+\sum_{j=1}^{3}\left(\varphi_{j\neq i}/\varrho_{j\neq i}\right)*100}{100\;-\;\left(\sum_{j=1}^{3}\left(\varphi_{i}/\varrho_{j\neq i}\right)/\sum_{j=1}^{3}\left(\varphi_{i}/\varrho_{j\neq i}\right)+\sum_{j=1}^{3}\left(\varphi_{j\neq i}/v_{i}\right)*100\right)}\\ \text{LNR}=\frac{100\;-\;\left(\varphi_{i}/\varrho_{i}/\varphi_{i}/\varrho_{i}+\sum_{j=1}^{3}\left(\varphi_{j\neq i}/\varrho_{j\neq i}\right)*100\right)}{\sum_{j=1}^{3}\left(\varphi_{i}/\varrho_{j\neq i}\right)/\sum_{j=1}^{3}\left(\varphi_{i}/\varrho_{j\neq i}\right)+\sum_{j=1}^{3}\left(\varphi_{j\neq i}/\varrho_{i}\right)*100}. \end{array}$$

Table 2 shows the proposed KNN and support vector machine model-based cancer, dementia, and diabetes prediction from multifactorial genetic inheritance disorder during the training phase. Total 1447 instances were used during training simulation; furthermore, these instances were divided into 103, 67, and 1276 sections of dementia, cancer, and diabetes, respectively. During the training session of the proposed SVM model, predicts 5, 57, 1268, 2, and 10 are correctly classified and 98 and 6 are wrongly classified. Similarly, the proposed KNN-based model training session predicts 2, 56, 1273, 1, and 11 are correctly classified and 101 and 2 are wrongly classified.

Table 3 shows the proposed support vector machine and KNN model-based cancer, dementia, and diabetes prediction from multifactorial genetic inheritance disorder during the testing phase. Total 620 instances were used during the testing phase; furthermore, these instances were divided into 49, 30, and 541 sections of dementia, cancer, and diabetes, respectively. During the testing phase of the proposed SVM model, predicts 3, 27, 541, and 3 are correctly classified and 46 is wrongly classified. Similarly, the proposed KNN-based model testing session predicts 6, 20, 530, and 10 are correctly classified and 43 and 11 are wrongly classified. It is observed that the proposed model of SVM has the highest correctly classified instances as compared with the proposed model of KNN.

**Figure 3:**
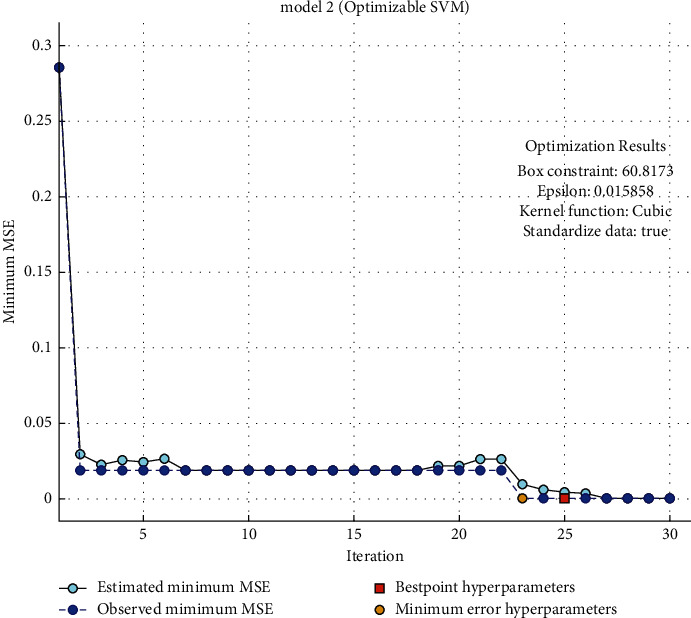
Performance of the proposed SVM-based model w.r.t MSSE vs. iterations.

Figure 3 shows the performance of the proposed SVM-based model with respect to minimum mean square error (MMSE) vs. iterations. It clearly observed that the proposed support vector machine-based model congregated at the 5th iteration with 0.00482 MMSE.

Table 4 shows the training simulation results of dementia class using different statistical parameters by the proposed model of SVM and KNN.  Table 5 shows the training simulation results of cancer class using different statistical parameters by the proposed model of SVM and KNN.  Table 6 shows the training simulation results of diabetes class using different statistical parameters by the proposed model of SVM and KNN.  Table 7 shows the testing simulation results of dementia class using different statistical parameters by the proposed model of SVM and KNN.  Table 8 shows the testing simulation results of cancer class using different statistical parameters by the proposed model of SVM and KNN.  Table 9 shows the testing simulation results of diabetes class using different statistical parameters by the proposed model of SVM and KNN.

Table 10 shows the number of performance statistical parameters used to calculate the performance of the proposed SVM and KNN prediction model to predict dementia, cancer, and diabetes from multifactorial genetic inheritance disorder.  Table 8 shows the statistical parameter like accuracy and miss-rate results of proposed SVM and KNN models, so the proposed SVM model achieves 92.8% and 7.2% of training accuracy and miss classification rate, respectively. Similarly, the proposed model of KNN achieves 92.8% and 7.2% of training accuracy and miss classification rate, respectively. In the prediction phase, the proposed SVM model achieves 92.5% and 7.5% of testing accuracy and miss classification rate, respectively, and the proposed KNN model achieves 91.2% and 8.8% of testing accuracy and miss classification rate, respectively.

Table 11 shows the comparative analysis of the proposed model with previous studies. As in Table 11, the proposed model outclassed all mentioned previous studies and achieved highest classification accuracy in all three diseases cancer, dementia, and diabetes as well as the proposed model of SVM for the prediction of dementia, cancer, and diabetes from multifactorial genetic inheritance disorder achieves the highest test classification accuracy as compared with the proposed model of KNN. The proposed model achieved highest prediction accuracy because it used different data preprocessing techniques to clean up the data and correlation techniques for extraction of highly relatable features, and the proposed model used all these features to predict the cancer, dementia, and diabetes.

## 7. Conclusion and Future Work

Machine learning plays a bigger role in the classification of different diseases in medical and biomedical fields. In this study, the proposed model used two machine learning techniques SVM and KNN to predict dementia, cancer, and diabetes from multifactorial genetic inheritance disorders. The proposed model analyzed the prediction results with respect to different statistical performance parameters. In this study, the proposed model used patients' multifactorial genetic inheritance disorder history to predict dementia, cancer, and diabetes because patient medical history puts a major impact on prediction results. The proposed model SVM achieves the highest testing prediction classification accuracy of 92.5% as compared with the proposed model of KNN. This study will play a major part in the medical field to early predict these dangerous diseases in the early stages of life with the help of the patient's genetic history and procure these diseases in early stages. Major advantage of this study is to predict the multiclass prediction of genome disorders, i.e., cancer, dementia, and diabetes with the help of major machine learning algorithms and correlation techniques. On the other hand, there will be more improvements in the light of data and other machine learning and transfer learning techniques. Furthermore, in the future, this study will expand to predict all these three diseases with the help of genetic sequence data and also, with the help of mitochondrial genetic inheritance disorder prediction of leigh syndrome and mitochondrial myopathy using machine learning techniques, federated machine learning and transfer learning will play a major role in the genetic field.

## Figures and Tables

**Table 1:** Description of dataset attributes.

No.	Attributes	Values
1	Patient age	0–16
2	Genes in mothers' side	1: yes; 2: no
3	Inherited from father	1: yes; 2: no
4	Maternal gene	1: yes; 2: no
5	Paternal gene	1: yes; 2: no
6	Gender	1: male; 2: female; 3: ambiguous
7	Birth asphyxia	1: yes; 2: no

**Table 2:** Training performance of the proposed three-class SVM and KNN-based model.

Instances (1447)	SVM			KNN		
	Dementia	Cancer	Diabetes	Dementia	Cancer	Diabetes
Dementia	5	0	98	2	0	101
Cancer	0	57	10	0	56	11
Diabetes	6	2	1268	2	1	1273

**Table 3:** Testing performance of the proposed three-class SVM and KNN-based model.

Instances (1447)	SVM			KNN		
	Dementia	Cancer	Diabetes	Dementia	Cancer	Diabetes
Dementia	3	0	46	6	0	43
Cancer	0	27	3	0	20	10
Diabetes	0	0	541	11	0	530

**Table 4:** Training simulation results of dementia class by the proposed model.

Instances (1446)	CA (%)	CMR (%)	Sensitivity (%)	Specificity (%)	F1-score (%)	NPV (%)	FPR (%)	FNR (%)	LPR (%)	LNR (%)	PPV (%)
SVM	94.77	5.23	45.45	95.16	11.9	99.5	4.84	54.55	9.39	0.57	6.84
KNN	94.84	5.16	50	92.99	3.73	92.99	7.01	50	7.13	0.53	1.94

**Table 5:** Training simulation results of cancer class by the proposed model.

Instances (1446)	CA (%)	CMR (%)	Sensitivity (%)	Specificity (%)	F1-score (%)	NPV (%)	FPR (%)	FNR (%)	LPR (%)	LNR (%)	PPV (%)
SVM	99.17	0.83	96.61	99.27	90.47	99.27	0.73	3.39	132.34	0.034	85.07
KNN	99.17	0.83	98.24	99.2	90.32	99.20	0.8	1.76	122.8	0.017	83.58

**Table 6:** Training simulation results of diabetes class by the proposed model.

Instances (1446)	CA (%)	CMR (%)	Sensitivity (%)	Specificity (%)	F1-score (%)	NPV (%)	FPR (%)	FNR (%)	LPR (%)	LNR (%)	PPV (%)
SVM	91.97	8.03	92.15	88.57	95.62	36.47	11.43	7.85	8.06	0.088	99.37
KNN	92.04	7.96	91.9	95.08	95.67	34.11	4.92	8.1	18.67	0.088	99.76

**Table 7:** Testing simulation results of dementia class by the proposed model.

Instances (620)	CA (%)	CMR (%)	Sensitivity (%)	Specificity (%)	F1-score (%)	NPV (%)	FPR (%)	FNR (%)	LPR (%)	LNR (%)	PPV (%)
SVM	92.58	7.42	100	92.54	12.24	100	7.46	0	13.4	0	6.12
KNN	91.29	8.71	35.29	92.86	18.18	98.07	7.14	64.71	4.94	0.69	12.24

**Table 8:** Testing simulation results of cancer class by the proposed model.

Instances (620)	CA (%)	CMR (%)	Sensitivity (%)	Specificity (%)	F1-score (%)	NPV (%)	FPR (%)	FNR (%)	LPR (%)	LNR (%)	PPV (%)
SVM	99.51	0.49	100	99.4	94.7	100	0.6	0	166.6	0	90
KNN	98.38	1.62	100	98.3	80	100	1.7	0	58.82	0	66.6

**Table 9:** Testing simulation results of diabetes class by the proposed model.

Instances (1446)	CA (%)	CMR (%)	Sensitivity (%)	Specificity (%)	F1-score (%)	NPV (%)	FPR (%)	FNR (%)	LPR (%)	LNR (%)	PPV (%)
SVM	92.09	7.91	91.69	100	95.66	37.97	0	8.31	0	0.083	100
KNN	89.67	10.33	90.90	70.27	94.30	32.91	29.73	9.1	3.05	0.129	97.96

**Table 10:** Proposed model parameter results.

Instances (2067)	SVM		KNN	
	Training (%) (1446 instances)	Testing (%) (620 instances)	Training (%) (1446 instances)	Testing (%) (620 instances)
Accuracy	92.8	92.5	92.8	91.2
Miss-rate	7.2	7.5	7.2	8.8

**Table 11:** Comparative analysis with previous work.

Work	Model	Dataset	Classification accuracy (%)
Kee Pang Soh et al. [20]	Logistic regression, random forest	Cancer mutate data	77.7
Javier De Velasco Oriol et al. [21]	Linear ML	SNP dataset	75
Bassam Farran et al. [22]	Logistic regression, KNN	Kuwait health network data	81.3
Proposed model for prediction of dementia, cancer, and diabetes	Machine learning (SVM and KNN)	Genome disorder data	92.5

## Data Availability

The data used in this paper can be obtained from the corresponding author upon request.
